# The Treatment of Chronic Otorreœa in School Children

**Published:** 1922-01

**Authors:** A. R. Friel

**Affiliations:** Aural Specialist, "Almeric Paget" and "Lissonia" Ionisation Clinics for Otorrhœa in School Children, London County Council; Aural Specialist, Ministry of Pensions Boards, London District


### THE TREATMENT OF CHRONIC OTORRHŒA IN SCHOOL CHILDREN.

By A. R. FRIEL, M.D., F.R.C.S.I., Aural Specialist, "Almeric Paget" and "Lissonia" Ionisation Clinics for Otorrhœa in School Children, London County Council; Aural Specialist, Ministry of Pensions Boards, London District.

WHEN a child suffering from chronic Otorrhœa is brought for treatment the first point which has to be elucidated is the "cause of the chronicity." There is certainly one factor, but there may be more, and success in treatment depends on discovering what is present.

Chronic suppurative otitis media is a sequel to acute otitis media, but the two diseases differ in their cause, and in the course they run. Acute otitis media is due to an attack on the tissues by one species of micro-organism, such as the pneumococcus, streptococcus or B. Influenzæ. In a few days, as a rule, the tissues develop sufficient bactericidal power to repel the organisms and the latter perish, provided one other factor is not introduced: viz., *infection of the discharge* by organisms from the skin, air, &c. Infection of the discharge results in the production of decomposing material outside the tissues, but in contact with them, which irritates them. The tissues respond by increased exudation of fluid and cells which are in turn decomposed, and so on, *ad infinitum.* This is the basic factor in the causation of chronicity. In stating this the author is applying Sir Almroth Wright's work on wounds to a special case of sepsis. Other factors may be superadded, such as the development of polypi or granulations, or the presence of caries.

The problem which it is necessary for us to solve, taking the simple, *i.e.*, uncomplicated, case first, is "How to disinfect the area occupied by the discharge without irritating the tissues forming the wall?"

Let us consider what we have at our disposal. First, we have as our greatest asset the protective and the bactericidal powers of the blood and tissues, but experience tells us that these by themselves are often not sufficient. External aid is required.

We know that moisture is essential to the life of bacteria; therefore if we remove the discharge and dry the cavity, and keep it dry, or dry it repeatedly, we shall succeed in removing the irritation and the discharge will cease. This is the principal way in which powders such as boracic acid act.

Again, if we could coat the surface of the mucous membrane with an impervious non irritating layer which would shut it off from external irritation we should limit the secretion of cells and fluid. This is the way in which alcohol acts. The layer of exudation on the surface of the mucous membrane or granulations is coagulated by the alcohol. The subjacent tissue is not irritated to any extent, partial rest is afforded to the tissues, and the discharge frequently ceases. The ear is not completely disinfected at once, for the action of the alcohol is superficial. There is not the penetration necessary to sterilise the ear completely.

Various antiseptics have been used with or without the alcohol. Their action is largely the sedative one in the mind of the doctor who prescribes them. All antiseptics like carbolic acid, hydrarg. perchlor., iodine, in strength sufficient to have an antiseptic action, are irritating, and in strength sufficient to be non-irritating are not antiseptic.

Lastly we may consider the action of the electric current on an ion such as zinc and the effect of the combination of these two on a septic ear. It is to Professor Leduc of Nantes that we owe our knowledge of the benefit of zinc ionisation in local sepsis.

Zinc ions coagulate albumen and the electric current makes the ions penetrate to any depth required. The coagulation of the albumen of cells, whether they are tissue cells or bacteria, involves their death. Thus the exudation is sterilised. The solution of the zinc salt can be used so weak that it does not irritate. The electric current takes the ions from a weak solution and deposits them homogeneously in the exudation as readily as from a strong solution.

Zinc ionisation furnishes a solution of the problem referred to above under "infection of the discharge," which has to be solved in all cases of chronic local sepsis.

(Cases of tuberculous disease are not inculded in the present discussion, as the tubercle bacillus is *in the tissues* as well as in the discharge.)

The coagulum that is formed is moreover a sterile barrier between the tissues and the exterior. Rest is afforded to the tissues, which rapidly progress towards structural integrity and functional efficiency.

The possibility of applying zinc ionisation successfully in the treatment of the individual case of chronic otorrhœa depends on:

1. The situation of the septic material.
2. The presence or absence of factors, additional to infection of the discharge, which favour the continuance of the otorrhœa.

These conditions may be stated in tabular form:

1. Tympanic conditions. *a*, Tympanic sepsis alone, or with granulations, polypi, or caries.
2. Tympanic conditions + Eustachian obstruction or infection from Septic Tonsils, Inflamed Adenoids, Rhinitis, Sinusitis, Septic Teeth.
3. Tympanic conditions + inflammation of the External Auditory Meatus.
4. Tympanic conditions + Mastoid or Attic Disease.

Chronic otorrhœa is due in many cases solely to the presence of septic material in the tympanum. If we remove the *macroscopic* mass from the cavity by syringing or swabbing and then sterilise the *microscopic* layer adhering to the surface, the irritation causing the discharge should be absent, the discharge should rapidly (usually immediately) and completely disappear, and such results should be consistently obtained In the writer's experience this is so in cases of simple tympanic sepsis when, as is usually the case, the perforation in the drum is large enough to

**The Treatment of Chronic Otorrhœa in School Children—(*cont.*).**

allow the middle ear to be filled with the zinc solution.

If there is much inflammatory swelling of the mucous membrane the probability is that the insufflation of some boracic acid powder immediately after ionisation, and perhaps at a later date, will be required to absorb mucous exudation taking place while the condition is subsiding. This should also be done when the meatal canal is inflamed.

If small granulations are present the insufflation of boracic powder after ionisation is sufficient to absorb and keep sterile the small amount of exudation from the granulations until in a few days these have disappeared.

If large granulations or polypi exist these require treatment prior to ionisation of the tympanum. The complicated condition should be converted into the simple one of tympanic sepsis alone.

An area of caries in the tympanum would prejudice the immediate and complete cessation of discharge.

It is not often possible to introduce the zinc solution into the mastoid antrum or cells, therefore in cases in which these are infected it is usually waste of time to use ionisation.

In the attic similar mechanical difficulties stand in the way, and an attic case is as a rule unfavourable.

Mere enlargement of the tonsils or the presence of adenoids is not a contraindication to treatment of the tympanum by ionisation. Purulent rhinitis, inflamed adenoids, or inflamed septic tonsils, much pyorrhœa, or nasal sinusitis are contraindications, because it is impossible to prevent reinfection of the tympanum under such circumstances.

It is of interest to know what proportion of cases of otorrhœa in children are readily amenable to zinc ionisation. From his experience the author concludes that in over 50 per cent. the otorrhœa can be readily cured.

A question which will be asked is : Are there many recurrences ? In the author's experience a recurrence is not common, but it should be remembered that zinc ionisation is not a method of producing immunity. Patients should be cautioned against bathing or allowing water to enter the ear. Anything which experience shows is likely to cause otorrhœa should be avoided. If a *large* mass of adenoids is present or the tonsils are greatly enlarged, we know that the owners are more liable to catarrh and, therefore, the former condition should be treated.

Otorrhœa is an excellent subject in which to learn the principles and practice of ionisation. Experience in treating it teaches that to be successful it is necessary to take into account the "topographical" factors as well as the electrical and physical ones involved. The amount of electrical knowledge necessary to practise ionisation is small and can soon be acquired. It takes much longer to learn to recognise the conditions present in the patients. Since these decide the question as to whether ionisation should be done, with or without some other treatment, such as removal of polypi, it is the author's conviction that the ionisation should be carried out by the surgeon in charge of the case. To send the patient to an electrotherapeutist who knows nothing about the ear, and who has no manipulative experience in dealing with ears, is to court failure. To accept responsibility and do the actual treatment oneself makes for success. It can be confidently affirmed that anyone who proceeds on these lines will have success.

In conclusion, a word may be said about what perhaps falls strictly under the head of organisation of a school clinic. The Doctor who carries out the treatment should examine *all* the cases of otorrhœa and decide on the cause of chronicity. If someone else selects the cases the probability will be that unsuitable ones are sent for ionisation, simply because they have not yielded to other treatment.

By deciding on the cause of chronicity in each individual case and treating it, in contradistinction to what is called "routine treatment," much time will be saved and disappointment avoided, and the success of the clinic will be assured.

---

The Medical Officer of Health for the City of London reports that of 48 samples of milk which arrived in the City, 12, or exactly 25 per cent., were infected with tubercle bacilli. The present method of eliminating infected milk from the public supply is, he considers, quite unsatisfactory. As the matter stands at present it has to be proved that the farmer is aware that he sells, or suffers to be sold or used for human consumption, the milk of a cow which is suffering from tuberculosis of the udder. Dr. Howarth said evidence to prove such a fact is difficult to obtain, and the responsibility of ensuring that the milk is free from tubercle ought to be cast directly on the farmer.

---

In connection with the Dentists Act, it is to be noted that the Dental Board authorised by that Act has now been appointed. Its personnel is as follows : Mr. F. D. Acland, M.P. (Chairman), Mr. Laurance George Brock, Mr. Fred Butterfield, Sir Arthur Gerald Chance, F.R.C.S. Irel., Mr. Dugald McCoig Cowan, M.P., Mr. William Henry Dolamore, M.R.C.S., L.R.C.P., L.D.S., Professor William Henry Gilmour, M.D.S., L.D.S., Mr. William Guy, F.R.C.S. Edin., L.R.C.P. Edin., L.D.S., Sir James Hodsdon, F.R.C.S. Edin., Mr. Horace Archibald Robertshaw, Mr. Edward Leo Sheridan, F.R.C.S. Irel., L.R.C.P. Irel., L.D.S., Mr. John Sinclair, Mr. Holburt Jacob Waring, F.R.C.S., and Mr. Norman C. King (Registrar). The offices of the Dental Board will be at 44, Hallam Street, Portland Place, London, W.1. All communications should be sent to the Registrar at that address.

---

The shortage of new-laid eggs at this time of the year inevitably leads to higher prices—the highest of the year; but Messrs. Wilkinson, our old advertisers, inform us that the tendency to the abnormal prices of the later war years has been checked. There are good supplies of new-laids from Holland and Denmark, and as there was a refusal to buy Irish at the rapidly increasing rates there has been a decided check. For instance, at Portadown, probably the best egg market in Ireland, prices went to 4s. 7d. per dozen for bare eggs. Add expenses and cases and carriage, and a cost of 5d. is nearly reached. Buyers fought shy, so prices dropped. The great feature, however, is the supply of really good "first" eggs from Canada, United States, Argentine, South Africa, Lithuania, Serbia, Russia, &c., so that for the many purposes for which really good eggs, though not new-laids, suffice there is a good supply, and that means that new-laids will not go dear. Messrs. Wilkinson tell us that by Christmas the new production will be well on the way, and rapidly falling prices by January. Hospital and nursing-home officials seeking good supplies of tested eggs should communicate with this firm of provision merchants, and perhaps the greatest egg experts.

